# The predictive factors of nocturia in young Asian adult males: an online survey

**DOI:** 10.1038/s41598-021-95836-4

**Published:** 2021-08-10

**Authors:** Weiming Cheng, Yu-Hua Fan, Ying-Jay Liou, Yi-Ting Hsu

**Affiliations:** 1 Division of Urology, Department of Surgery, Taipei City Hospital, Zhongxiao Branch, Taipei, Taiwan; 2 Department of Urology, Taipei Veterans General Hospital, Taipei, Taiwan; 3 Department of Psychiatry, Taipei Veterans General Hospital, Taipei, Taiwan; 4 Division of Urology, Department of Surgery, Taipei City Hospital, Renai Branch, No. 10, Sec. 4, Renai Rd., Daan Dist., Taipei, 106243 Taiwan; 5 Program in Molecular Medicine, College of Life Science, National Yang Ming Chiao Tung University, Taipei, Taiwan; 6 Institute of Biopharmaceutical Science, National Yang Ming Chiao Tung University, Taipei, Taiwan; 7 Department of Urology, College of Medicine, National Yang Ming Chiao Tung University, Taipei, Taiwan; 8 Department of Psychiatry, College of Medicine, National Yang Ming Chiao Tung University, Taipei, Taiwan

**Keywords:** Physiology, Psychology, Health care, Risk factors, Urology

## Abstract

The present study investigated the association between severity of depressive mood and nocturia in young Asian adult men. Participants were 3127 adult male Facebook users aged 20–40 years who could read and write traditional Chinese. Participants completed online questionnaires on demographic characteristics, frequency of waking to urinate during the night (International Prostate Symptoms Score [IPSS]), and frequency of depressive symptoms (Taiwanese Depression Questionnaire [TDQ]). Those who awoke to pass urine during the main sleep period were considered to have nocturia. Student’s t test and Pearson’s chi square test were used to compare participants with and without nocturia. Univariate and multivariate logistic regression were used to evaluate predictive factors for nocturia. One thousand four hundred thirty (45.7%) participants had nocturia, and 21.9% were suspected to have depression. Age over 30 years, body mass index over 25 kg/m^2^, and higher IPSS score (except times of nocturnal voiding) were factors predictive of nocturia. Higher TDQ somatic subscores, rather than affective/cognitive subscores, were also predictive of nocturia. Associations were found between normal-high TDQ scores and nocturia. Young men with nocturia at risk of developing depression should be identified with a culturally relevant questionnaire. Early referral for psychiatric assessment and intervention may be warranted.

## Introduction

Nocturia is highly prevalent in the general population. It causes sleep disturbance and exerts a profound impact on quality of life. Not only is it the main lower urinary tract symptom among older adults, but it is also common in young adults^1^. According to the Boston Area Community Health Study, 19.9% of the residents aged between 30 and 39 years complained of nocturia^2^. Medical comorbidities, such as benign prostate hyperplasia and heart failure, would induce or exacerbate nocturia. Nevertheless, these medical conditions are infrequent in young adults. On the other hand, psychological stress and psychiatric problems, particularly depression, would be the main risk factor for nocturia^3^. A systematic review of seven studies suggests that there is a strong connection between depression and the incidence of nocturia, especially in men^4^. However, none of the studies have investigated the association between nocturia and depression in younger populations. Moreover, given the possible differences in the expression of depression in different cultures^5^, it would be important to investigate whether the relationship exists in Asian populations. Therefore, in this study, an online survey was conducted to investigate the association between the severity of depressive mood and nocturia in young Asian adult men.

## Materials and methods

### Questionnaire

An online questionnaire was designed in traditional Chinese to investigate the relationship between nocturia and the degree of depressive symptoms. The first part comprised questions about participant demographics, including age, body height, and body weight. In the second part, the 7-item International Prostate Symptoms Score (IPSS) was used to evaluate the severity of lower urinary tract symptoms (LUTS). The IPSS can range from 0 to 35, and scores are categorized as mild (0–7), moderate (8–19), and severe (20–35). Subscores can be calculated for voiding (4 items, range = 0–20) and storage (3 items, range = 0–15). The number of times the participant awoke to urinate during nighttime sleep was obtained from the question, “Over the past month, how many times did you most typically get up to urinate from the time you went to bed at night until the time you got up in the morning?” Waking to pass urine during the main sleep period was considered an indication of nocturia, according to the International Continence Society (ICS) 2018 definition^6^. The third part was the Taiwanese Depression Questionnaire (TDQ), which was used to assess the level of depression. The TDQ is a culturally-relevant questionnaire composed of 18 questions regarding the frequency of somatic symptoms and emotional distress within the past week. The response scale is as follows: 0 = fewer than one day per week, 1 = 1–2 days per week, 2 = 3–4 days per week, and 3 = 5–7 days per week. The total score ranges from 0 to 54. The questionnaire has been validated with a sensitivity of 0.89 and specificity of 0.92, at a cutoff score of 19^7^. The scores on the TDQ can be used to classify individuals into four groups: normal (score 0–8), normal-low (score 9–14), normal-high (score 15–18), and depression (score ≥ 19)^8^. The 18 items are either affective/cognitive questions or somatic questions; therefore, an affective/cognitive symptoms subscore and a somatic symptoms subscore were calculated for further analysis^8^.

### Study design

The study was approved by the Institutional Review Board of Taipei City Hospital (TCHIRB-10911002-E). The online questionnaire was administered on SurveyCake, a commercialized software website for creating customized survey questionnaires. Adult male Facebook users aged between 20 and 40 years who could read and write traditional Chinese were invited to participate in the study by providing a hyperlink on Facebook to the questionnaire. The online questionnaire was available from February 1st to 28th, 2021. Refilling was prevented by a check question at the beginning of the questionnaire and by Internet protocol address filtering.

### Statistical analysis

Statistical analysis was performed using IBM SPSS Statistics for Mac, ver. 24 (IBM Corp., Armonk, NY, USA). All data were expressed as the percentage or mean ± standard deviation. Student’s t test and Pearson’s chi square test were used to compare the differences between participants with and without nocturia, depending on continuous or categorical parameters. Univariate and then multivariate logistic regression were used to evaluate the predictive factors for nocturia. A p value less than 0.05 was considered statistically significant.

### Ethics statement

All procedures were in accordance with the ethics committee and with the principles of the Declaration of Helsinki. The ethics committee confirmed that no written informed consent was warranted from participants included in this study.

## Results

A total of 3,127 Asian adult males completed the questionnaire online. Table 1 shows the demographic characteristics of the participants. One thousand four hundred thirty (45.7%) participants fulfilled the criteria for nocturia. The mean IPSS of all participants was 5.2 ± 5.2. Most of the participants (75.6%) were classified as having mild LUTS, and 22.0% and 2.4% had moderate and severe LUTS, respectively. The mean TDQ score was 11.9 ± 10.1. Of all the participants, 21.9% were suspected to have depression (TDQ score ≥ 19).

**Table 1:** Characteristics of total participants and participants with and without nocturia.

Characteristic	Total *N* = 3127		No nocturia *N* = 1697		With nocturia *N* = 1430		*p* value
Age: years (*SD*)	30.7	(5.5)	29.6	(5.4)	32	(5.3)	< .001
BMI: kg/m^2^ (*SD*)	25	(4.6)	24.5	(4.4)	25.5	(4.8)	< .001
IPSS (*SD*)	5.2	(5.2)	3.7	(4.2)	7.1	(5.7)	< .001
Voiding score (*SD*)	2.6	(3.4)	2.0	(2.9)	3.3	(3.8)	< .001
Storage score (*SD*)	2.7	(2.4)	1.7	(1.9)	3.8	(2.5)	< .001
IPSS except times of nocturnal voiding (*SD*)	4.7	(4.9)	3.7	(4.2)	5.9	(5.5)	< .001
IPSS							< .001
Mild LUTS: no. (%)	2365	(75.6%)	1447	(85.3%)	918	(64.2%)	
Moderate LUTS: no. (%)	687	(22.0%)	235	(13.8%)	452	(31.6%)	
Severe LUTS: no. (%)	75	(2.4%)	15	(0.9%)	60	(4.2%)	
TDQ Score (*SD*)	11.9	(10.1)	10.9	(9.7)	13.1	(10.5)	< .001
Depression (TDQ score ≥ 19)							< .001
Yes: no. (%)	684	(21.9%)	329	(19.4%)	355	(24.8%)	
No: no. (%)	2443	(78.1%)	1368	(80.6%)	1075	(75.2%)	
TDQ subscore							
Affective/cognitive score (*SD*)	7.4	(6.7)	6.8	(6.5)	8	(6.9)	< .001
Somatic score (*SD*)	4.5	(3.9)	4.1	(3.7)	5.1	(4)	< .001
TDQ category							< .001
Normal: no. (%)	1432	(45.8)	844	(49.7)	588	(41.1)	
Normal-low: no. (%)	680	(21.7)	366	(21.6)	314	(22)	
Normal-high: no. (%)	331	(10.6)	158	(9.3)	173	(12.1)	
Depression: no. (%)	684	(21.9)	329	(19.4)	355	(24.8)	

BMI = body mass index; IPSS = International Prostate Symptoms Score; LUTS = lower urinary tract symptoms; TDQ = Taiwanese Depression Questionnaire.

Participants with nocturia were older (32 ± 5.3 years vs. 29.6 ± 5.4 years, *p* < 0.001), more overweight (BMI: 25.5 ± 4.8 kg/m^2^ vs. 24.5 ± 4.4 kg/m^2^, *p* < 0.001), and had a higher IPSS (except times of nocturnal voiding) (5.9 ± 5.5 vs. 3.7 ± 4.2, *p* < 0.001), as well as higher voiding and storage subscores (Table 1). They also had higher mean total TDQ scores (13.1 ± 10.5 vs. 10.9 ± 9.7, *p* < 0.001) and affective/cognitive and somatic subscores.

In the univariate analysis, all the parameters were significantly associated with nocturia (Table 2). Three multivariate analysis models with different parameters were used to evaluate the effect of depression on nocturia. Age over 30 years, BMI over 25 kg/m^2^, and IPSS score (except times of nocturnal voiding) were predictive factors for nocturia in all three models. With regard to depressive symptoms, in the first model, depression based on TDQ score (≥ 19) was not a significant predictor of nocturia (odds ratio 1.045, 95% confidence interval [0.869 1.257], *p* = 0.64) (Table 2). In the second model, only TDQ somatic subscores (odds ratio 1.042, 95% confidence interval [1.008 1.078], *p* = 0.01) predicted nocturia. In the third model, normal-high depressive mood rather than depression was a significant predictor of nocturia, when compared to a normal mood. An analysis was also conducted to examine the depressive symptoms predictive of nocturia, based on the questions listed in the TDQ. Questions related to crying, irritability, poor sleep, chest tightness, tiredness, and body discomfort were associated with nocturia in young Asian males after adjustment with age, BMI, and IPSS (except times of nocturnal voiding) (Table S1). Among the somatic depressive symptoms, only the question with reference to poor sleep (odds ratio 1.103, 95% confidence interval [1.003 1.212], *p* = 0.042) was significantly predictive of nocturia.

**Table 2:** Results of the univariate analysis and three multivariate analyses.

Characteristic	Univariate analysis		Multivariate analysis model I		Multivariate analysis model II		Multivariate analysis model III	
	Odds ratio	*p* value	Odds ratio	*p* value	Odds ratio	*p* value	Odds ratio	*p* value
**Age (years)**								
< 30	1 (ref)		1 (ref)		1 (ref)		1 (ref)	
> 30	2.12	< .001	2.009	< .001	2.012	< .001	2.016	< .001
**BMI (kg/m**^**2**^**)**								
< 25	1 (ref)		1 (ref)		1 (ref)		1 (ref)	
> 25	1.43	< .001	1.392	< .001	1.393	< .001	1.392	< .001
IPSS except nocturia	1.099	< .001	1.095	< .001	1.088	< .001	1.092	< .001
**Depression based on TDQ**								
TDQ score < 19	1 (ref)		1 (ref)					
TDQ score ≥ 19	1.373	< .001	1.045	.64				
**TDQ subscore**								
Affective/cognitive	1.027	< .001			0.99	.29		
Somatic	1.065	< .001			1.042	.01		
**TDQ category**								
Normal	1 (ref)						1 (ref)	
Normal-low	1.231	.02					1.118	.25
Normal-high	1.572	< .001					1.302	.04
Depression	1.549	< .001					1.127	.24

BMI = body mass index; TDQ = Taiwanese Depression Questionnaire; IPSS = International Prostate Symptoms Score.

## Discussion

Most previous studies on nocturia have focused on men older than 50 years. For this purpose, the present study recruited younger men who have rarely been investigated in the literature. Moreover, this study excluded causes of nocturia from organic diseases of the lower urinary tract, such as benign prostate hyperplasia, which are infrequent among younger men. Results of the online questionnaire survey revealed that the prevalence of nocturia in Asian adult males younger than 40 years of age was 45.7%. This finding is consistent with other studies. The reported prevalence rate of nocturia in young males aged 20 to 40 ranged from 11 to 35.2%^9^. Another recent study showed the estimated prevalence of nocturia was 56.8% among young men in the U.S.,^10^. Thus, the sample of the present study, recruited from Facebook, could be representative of younger men in the general population.

Other studies including younger and older adults have revealed that increased age and BMI were both risk factors for nocturia. Madhu et al.^11^ conducted a secondary analysis of the EpiLUTS data using participants with nocturia. Age, BMI, anxiety, depression, and a history of bed-wetting were significantly associated with nocturia. Fitzgerald et al.^2^ found that the odds ratio of nocturia increased with age and BMI in a multivariate model. Other studies also reported that nocturia was significantly associated with obesity^12,13^. Similarly, in the present study, age older than 30 years, BMI over 25 kg/m^2^, and higher IPSS score (except times of nocturnal voiding) were predictive factors for nocturia in the sample of young Asian men. Goessaert et al.^14^ found that reduced functional bladder capacity was associated with nocturia in younger participants, which deteriorated with aging. Obesity increased intra-abdominal pressure and led to nocturia^15^. In addition, those with obesity had a higher chance of obstructive sleep apnea, which resulted in nocturnal polyuria and detrusor instability^16,17^. One supportive evidence in this study was that the participants with nocturia had a higher IPSS (except times of nocturnal voiding) score, especially with regards to the storage scores. This relationship has been found to be more profound in men^18^ and younger patients^19,20^, similar to the present study’s population.

One of the key findings of the present study was the association between nocturia and level of depression in young men, which differed from previous findings. Asplund et al.^21^ found that major depression was associated with a six-fold increase in nocturia in men. A cohort study by Johnson et al.^22^ showed that depressed and nondepressed patients reported a mean of 2.7 and 1.9 episodes of nocturia per night, respectively. Patients with five or more episodes of nocturia per night experienced a 6.5-fold increased risk of depression. Häkkinen et al.^3^ noted a unidirectional effect of depressive symptoms on the incidence of moderate or severe nocturia. A systematic review concluded a bidirectional association between depression and nocturia: nocturia increased the odds ratio of depression (OR 1.2–20.24), while depression similarly increased the odds ratio of nocturia (OR 1.2–7.73)^4^. The findings of this study indicated that depression based on TQD criteria was not associated with nocturia in young Asian men. Instead, young men with normal-high depressive mood were at risk of having nocturia. One possible explanation is that this study only focused on young men aged between 20 and 40 years, which is different from other studies that included men of both young and old ages.

There are several potential mechanisms underlying the relationship between depression and nocturia. One possibility is through poor sleep quality. Nocturia has been shown to have close relationships with early waking and decreased total sleep time^23^. In a cohort of patients treated for depression, depression severity significantly correlated with sleep quality^24^. Przydacz et al.^25^ even argued that it was not depression severity, but rather sleep quality that correlated with nocturia. The present study also found that poor sleep was the only somatic symptom in TDQ scores significantly predictive of nocturia. Second, depression has been shown to have a negative effect on perception, development, and prolongation of LUTS, including nocturia^26^. Third, depression may involve both increased nocturnal diuresis via a disturbed 24-h rhythm of antidiuretic hormone secretion, and a decrease in nocturnal bladder capacity through a central and/or peripheral serotonergic effect^21^. Fourth, in a previous study^27^, patients being treated with selective serotonin-reuptake inhibitors for depression had twice the incidence of nocturia. Another study^28^ also showed that the incidence and severity of overactive bladder, whose symptoms include nocturia, increased in men using antidepressants.

The present study found that somatic rather than affective/cognitive symptoms were related to nocturia, especially poor sleep, chest tightness, tiredness, and body discomfort. One possible explanation is the different expression of depression in different cultures. For example, Vietnamese patients were prone to endorse higher levels of somatic symptoms than German patients despite similar levels of depression severity^29^. Similarly, somatic complaints predicted depression in Vietnamese and Vietnamese American adolescents, whereas no relationship was found in European American adolescents^30^. Furthermore, only normal-high depressive mood could predict the presence of nocturia among young Asian men. Thus, the complaints of nocturia in young Asian males could be a red flag signaling a depressive mood, which could progress to major depressive disorder without early intervention. Therefore, by utilizing a culturally-relevant depression screening instrument, such as the TDQ, with young male patients complaining of nocturia in urology clinics, early identification of those who are at risk of developing a depressive disorder would be possible.

There are several limitations to this study that should be mentioned. First, this study recruited participants from a social media site (i.e., Facebook) as opposed to a community-based population. As such, selection bias may exist. It is reported that social media use was significantly associated with, or even causal of depression among young adults, especially among problematic users^31–33^. Nighttime-specific social media users experienced lower self-esteem, higher levels of anxiety and depression, and poorer sleep quality^34^, which may also impact the occurrence of nocturia. However, 98–99% of Taiwanese aged between 20 and 40 are Internet users, and therefore participant recruitment from social media may be feasible for studies involving young adults. Second, this study did not investigate comorbidities, medical history, diet, physical activity, or psychiatric history of the participants, which may also have an influence on the occurrence of nocturia. Nevertheless, it focused on young men instead of older adult men; it was posited that they would have less comorbidities and medications related to nocturia, and therefore, less confounding factors. Third, the information from a 24-h voiding diary, which could help determine the etiology of nocturia, is nearly impossible to obtain from an online survey. Nevertheless, there are some advantages of the present study. Over 3,000 young men were recruited; a population that has been less investigated for LUTS including nocturia. This study also evaluated the associations between depression and nocturia with a culturally-relevant questionnaire. The findings of the present study have practical implications for urologists when treating young men with nocturia. Early assessment of depressive symptoms can be preventative by enabling referrals for psychological evaluation and treatment of psychological symptoms.

## Conclusion

This study demonstrated that about 45.7% of men between the ages of 20 and 40 had nocturia. Age over 30 years, BMI over 25 kg/m^2^, and higher IPSS score (except times of nocturnal voiding) were independently predictive of nocturia. Especially, those with normal-high depressive mood (TDQ score 15–18) were also at risk of having nocturia. Because the expression of depression in people from Eastern cultures may be predominantly somatic in nature, it would be imperative to use a culturally-relevant questionnaire to uncover the potential psychosomatic connection between nocturia and depression in young men. Early referral of young men with nocturia for psychiatric assessment may be warranted.

## Supplementary Information

Supplementary Information.
