# Supplementary material for: The predictive factors of nocturia in young Asian adult males: an online survey
Source: Sci Rep. 2021 Aug 10;11:16218. doi: 10.1038/s41598-021-95836-4 (PMC8355330; doi:10.1038/s41598-021-95836-4)
Supplement: Supplementary file 1 — Supplementary Information. [file 41598_2021_95836_MOESM1_ESM.docx]

**The Predictive Factors of Nocturia in Young Asian Adult Males – An Online Survey**

Weiming Cheng, Yu-Hua Fan, Ying-Jay Liou, Yi-Ting Hsu

**Table S1.** Odds Ratio of Each Question from TDQ Questionnaire to Predict Nocturia by Logistic Regression

|  |  | Logistic regression |  | Logistic regression adjusted with age, BMI, and IPSS (except times of nocturnal voiding) |  |
| --- | --- | --- | --- | --- | --- |
| Question | Question type | Odds ratio (95% CI) | *p value* | Odds ratio (95% CI) | *p value* |
| 1 Crying | A/C | 1.267 (1.108, 1.449) | <.001 | 1.15 (1.002,1.336) | .047 |
| 2 Sadness | A/C | 1.207 (1.104,1.32) | <.001 | 1.097 (0.995,1.208) | .062 |
| 3 Irritability | A/C | 1.258 (1.152,1.374) | <.001 | 1.134 (1.032,1.246) | .009 |
| 4 Poor sleep | S | 1.276 (1.181,1.378) | <.001 | 1.154 (1.062,1.254) | <.001 |
| 5 Poor appetite | S | 1.054 (0.927,1.199) | .42 | 1.002 (0.872,1.151) | .981 |
| 6 Chest tightness | S | 1.416 (1.258,1.594) | <.001 | 1.204 (1.061,1.366) | .004 |
| 7 Uneasiness | A/C | 1.19 (1.089,1.3) | <.001 | 1.051 (0.955,1.157) | .311 |
| 8 Tiredness | S | 1.275 (1.174,1.384) | <.001 | 1.136 (1.039,1.242) | .005 |
| 9 Restlessness | A/C | 1.169 (1.078,1.267) | <.001 | 1.066 (0.976,1.163) | .155 |
| 10 Poor memory | S | 1.175 (1.088,10268) | <.001 | 1.024 (0.942,1.113) | .576 |
| 11 Concentration difficulty | S | 1.15 (1.059,1.248) | <.001 | 1.017 (0.928,1.113) | .723 |
| 12 Indecisiveness | A/C | 1.21 (1.111,1.319) | <.001 | 1.059 (0.964,1.163) | .233 |
| 13 Loss of self-confidence | A/C | 1.14 (1.059,1.227) | <.001 | 1.03 (0.951,1.116) | .47 |
| 14 Pessimism | A/C | 1.172 (1.089,1.261) | <.001 | 1.081 (0.998,1.171) | .056 |
| 15 Suicidal thought | A/C | 1.154 (1.037,1.284) | .009 | 1.034 (0.921,1.161) | .568 |
| 16 Loss of interest | A/C | 1.185 (1.081,1.298) | <.001 | 1.029 (0.931,1.138) | .574 |
| 17 Body discomfort | S | 1.344 (1.219,1.481) | <.001 | 1.153 (1.038,1.282) | .008 |
| 18 Worthlessness | A/C | 1.087 (1.007,1.173) | .033 | 0.989 (0.91,1.076) | .802 |

S=somatic; A/C=affective/cognitive; CI = confidence interval; IPSS = International Prostate Symptom Score
